# Area Deprivation Index and Rurality in Relation to Lung Cancer Prevalence and Mortality in a Rural State

**DOI:** 10.1093/jncics/pkaa011

**Published:** 2020-03-07

**Authors:** Kathleen M Fairfield, Adam W Black, Erika C Ziller, Kimberly Murray, F Lee Lucas, Leo B Waterston, Neil Korsen, Darlene Ineza, Paul K J Han

**Affiliations:** p1 Center for Outcomes Research and Evaluation, Maine Medical Center Research Institute, Portland, ME 04101, USA; p2 Muskie School of Public Service, University of Southern Maine, Portland, ME 04101, USA; p3 Bowdoin College, Brunswick, ME 04011, USA

## Abstract

**Background:**

We sought to describe lung cancer prevalence and mortality in relation to socioeconomic deprivation and rurality.

**Methods:**

We conducted a population-based cross-sectional analysis of prevalent lung cancers from a statewide all-payer claims dataset from 2012 to 2016, lung cancer deaths in Maine from the state death registry from 2012 to 2016, rurality, and area deprivation index (ADI), a geographic area-based measure of socioeconomic deprivation. Analyses examined rate ratios for lung cancer prevalence and mortality according to rurality (small and isolated rural, large rural, or urban) and ADI (quintiles, with highest reflecting the most deprivation) and after adjusting for age, sex, and area-level smoking rates as determined by the Behavioral Risk Factor Surveillance System.

**Results:**

Among 1 223 006 adults aged 20 years and older during the 5-year observation period, 8297 received lung cancer care, and 4616 died. Lung cancer prevalence and mortality were positively associated with increasing rurality, but these associations did not persist after adjusting for age, sex, and smoking rates. Lung cancer prevalence and mortality were positively associated with increasing ADI in models adjusted for age, sex, and smoking rates (prevalence rate ratio for ADI quintile 5 compared with quintile 1 = 1.41, 95% confidence interval [CI] =1.30 to 1.54) and mortality rate ratio = 1.59, 95% CI = 1.41 to 1.79).

**Conclusion:**

Socioeconomic deprivation, but not rurality, was associated with higher lung cancer prevalence and mortality. Interventions should target populations with socioeconomic deprivation, rather than rurality per se, and aim to reduce lung cancer risk via tobacco treatment and control interventions and to improve patient access to lung cancer prevention, screening, and treatment services.

Lung cancer is the leading cause of cancer death in the United States among both men and women, with 142 670 deaths estimated in the United States for 2010 (1). Efforts to reduce lung cancer mortality have focused on improving prevention via tobacco treatment and control, as well as access to early detection and treatment. Targeting such efforts to populations with the highest disease prevalence and mortality may improve their effectiveness.

Residents of rural areas are at greater risk for lung cancer incidence and mortality than their urban counterparts, and these disparities have been growing over time as urban declines in incidence and mortality have outpaced rural declines (2). Furthermore, although incidence rates of most cancers are lower in rural vs urban areas, crude mortality rates are higher in rural areas (2). Multiple studies have attempted to determine whether rural-urban disparities in cancer incidence and higher mortality is explained by differences in stage at diagnosis and/or treatment modality but have yielded mixed results (3). A recent study using the North American Association of Central Cancer Registries found that rural residents had lower total rates of localized cancers and higher total rates of advanced-stage cancers, suggesting possible rural-urban differences in cancer screening and detection (4). Rural rates of lung cancer incidence were higher at both localized and advanced stages, suggesting a greater absolute burden of lung cancer in rural vs urban areas (4).

Yet the reasons for rural-urban disparities in cancer incidence and mortality remain unclear. For example, rural residents are also more likely to smoke than their urban counterparts, a trend that has been increasing over time (5). Because smoking is the primary risk factor for lung and other cancers and is also associated with poorer survival (6), rural-urban disparities in smoking may drive disparities in both cancer incidence and mortality. Rural-urban disparities may also be driven by travel distance from urban centers or other geographic factors. Residents of rural areas also have limited access to health care, because of fewer primary and specialty care providers per capita (7) and, more recently, increasing rates of hospital closures (8).

Alternatively, rural-urban disparities in cancer incidence and mortality may be driven primarily by socioeconomic factors—specifically, the generally poorer economic circumstances of rural populations. A body of literature indicates that individual-level and area-level socioeconomic factors such as lower income and education are associated with higher cancer incidence and mortality, particularly for lung, cervical, stomach, and liver cancer (9). Hypothesized mechanisms explaining these associations between socioeconomic factors and cancer mortality include lack of insurance coverage, transportation, and other barriers to accessing quality cancer treatment. The negative influence of these factors on health has been usefully characterized through the concept of socioeconomic deprivation (9).

Socioeconomic deprivation may account for the observed rural-urban disparities in cancer outcomes. At the individual and population level, socioeconomic deprivation has been associated with behavioral risk factors for cancer such as smoking, physical inactivity, poor diet, and alcohol use (9) and may help explain rural-urban disparities in lung cancer incidence and survival. Rural residents are more likely than urban residents to report low income and education (7), to be uninsured or underinsured (7,10,11), and to delay using health-care services because of costs (12-14). At the community level, 30% of residents of rural zip codes report severe economic distress compared with only 15% of residents of urban zip codes (15).

The unresolved question is whether the disparately high cancer incidence and mortality among residents of rural vs urban areas is driven more by geographic as opposed to socioeconomic factors. Singh et al. (16) reported independent relationships between both rurality and socioeconomic deprivation and lung cancer mortality and found that deprivation was more strongly related to mortality than rurality. However, additional research is necessary to understand the relative magnitude of these effects and the underlying factors, such as smoking, that might influence these relationships.

The purpose of our study was to address this need by examining and comparing the independent associations between both rurality and socioeconomic deprivation and cancer prevalence and mortality. To this end, we focused on lung cancer and analyzed data from a predominantly rural state with high deprivation and a substantial lung cancer burden. We measured socioeconomic deprivation using the area deprivation index (ADI), a composite measure of socioeconomic deprivation in geographic regions. This measure is becoming more widely used and has been used to demonstrate that deprivation is associated with higher rates of 30-day rehospitalization (17), childhood asthma (18), and diabetes prevalence (19), as well as adverse health outcomes, including 30-day hospital readmission (17). By studying both rurality and deprivation simultaneously, we sought to understand the extent to which rural-urban differences in lung cancer burden are better explained by geographic or socioeconomic features of rural communities.

## Methods

We conducted a population-based cross-sectional analysis of: prevalent lung cancers identified in a statewide multipayer claims dataset, lung cancer deaths in Maine ascertained by the state death registry, rurality, and ADI. Analyses examined rate ratios for lung cancer prevalence and mortality according to rurality (small and isolated rural, large rural, or urban) and ADI (in quintiles, with highest reflecting the most deprivation). The variables that were measured and analyzed were derived from several different data sources. The Maine Medical Center Human Subjects Committee approved this research.

### Lung Cancer Prevalence

Prevalent lung cancers were identified from a multipayer medical claims dataset from 2012 to 2016, the most recent data available at the time of this analysis. This data includes insurance claims and eligibility information for nearly all beneficiaries in Maine insured by Medicare, Medicaid, or commercial insurers. We excluded patients who were younger than 20 years of age at diagnosis, were not insurance-eligible in the time period of observation, or did not have a zip code on file. Current International Classification of Disease (ICD)-9 and -10 codes were used to identify all prevalent lung cancer cases represented in the data (Supplementary Table 1, available online). It was required that a lung cancer diagnosis was given on an inpatient hospital claim, two hospital outpatient claims separated by 7 or more days, two office visit evaluation and management claims separated by 7 or more days, or one hospital outpatient and one evaluation and management separated by 7 or more days. We de-duplicated the cases, and each individual person only appears as one case of prevalent lung cancer over all of the years of data. After applying exclusions, 1 223 006 patients were included in the claims. Patients contributed person-time to the numerator during months when they were included in an eligibility file, and this was rolled up to person-years for prevalence analyses.

### Lung Cancer Mortality

Maine state death certificate data files for adults were obtained for 2012-2016 and included zip code of residence at the time of death, age, sex, and ICD-9 and -10 code for underlying cause of death. We used definitions from the Centers for Disease Control and Prevention (CDC) to aggregate ICD-9 and -10 codes for counts of deaths attributable to lung cancer (18). We did not attempt to ascertain or confirm deaths using claims data, and some deaths may have been from cases that did not appear in the claims files.

### Population Size

We used 2010 US Census data for Maine to calculate person-year denominators for all study outcomes. Because we examined 5 years of data for each outcome, the 2010 census counts for residents aged 20 years and older by age group and sex were multiplied by 5 to create a person-year denominator for prevalence and mortality.

### Smoking Prevalence

Smoking prevalence was estimated using Maine’s Behavioral Risk Factor Surveillance System (BRFSS) survey data obtained through the Maine CDC. The data include age and zip code of residence at the time of the survey. Data are collected by the CDC via stratified telephone sampling and weighted to be representative of the adult Maine population, aged 18 years and older (19). Data were aggregated over 4 years (2011-2015) to increase the stability of rate estimates. Sampling weights provided by the CDC were used to estimate percentage of ever-smokers in Maine according to cut points for rurality and ADI described above (20). Respondents were considered ever-smokers if they responded positively to a question about smoking at least 100 cigarettes during their lifetime. We had smoking data on 37 941 respondents during this time period.

### Rurality and Socioeconomic Deprivation

Rurality was measured using federal rural-urban commuting area (RUCA) codes, which are census-tract measures based on a combination of urbanicity, population density, and commuting patterns. We selected RUCA codes because many other measures are county-based and may be less precise at identifying rural communities, particularly in states with geographically large counties like Maine (21). We used patient zip codes from their claims to assign patients to RUCA codes using the RUCA zip code approximation file that crosswalks the RUCA census tracts to zip codes (21,22). The 10-level RUCA code was categorized into three groups: small and isolated rural (<10 000 population), large rural (“micropolitan,” 10 000-49 999), or urban (“metropolitan,” ≥50 000) (22).

Socioeconomic deprivation was measured using the ADI, a composite index of socioeconomic status (SES) calculated at the census block group level using 17 measures of poverty, education, housing, and employment indicators, using data from the 2013 American Community Survey (23). We then assigned ADI to five-digit zip codes because this was the only geographic variable available for this analysis. ADI category was collapsed into quintiles for this analysis.

### Statistical Analyses

**Figure 1.**
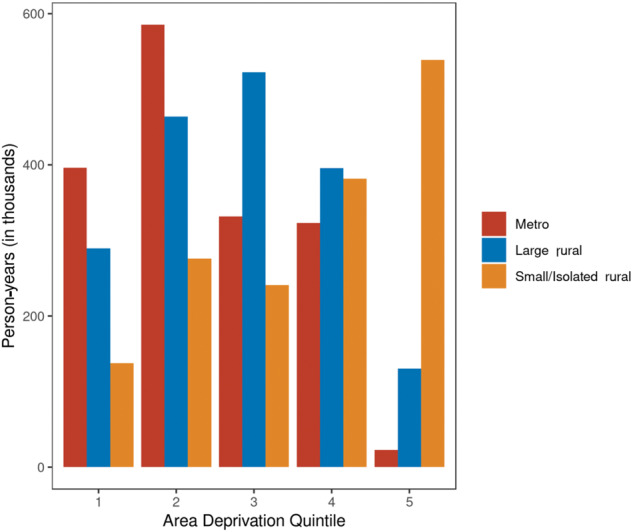
Relationship between rurality and area deprivation index across person-years, in thousands.

Descriptive statistics were obtained to examine the distribution of study variables and to explore geographic differences among them, including smoking rates by rurality and ADI. Predicted probability of having ever smoked given age, rurality, and area deprivation group was derived from the BRFSS data using logistic regression. Poisson regression analyses, both unadjusted and adjusted for age, sex, and smoking prevalence, were used to examine the independent associations between lung cancer prevalence and mortality and both rurality and ADI. The unit of observation was the age, sex, or smoking level stratum for each combination of covariates. Additionally, we conducted regression analyses including both rurality and ADI to assess their joint effects. We also characterized person-years as distributed across ADI categories and rurality (Figure 1). All statistical analyses were done using R version 3.5.1. The *P* values reported are derived from a test of linear contrast (a two-sided statistical test) from the Poisson models, and we used the cut point for statistical significance of *P* less than .05.

## Results

Among 1 253 011 adults aged 20 years and older in the all-payer dataset during the 5-year observation period, 8287 received care for lung cancer between 2012 and 2016 (Table 1). Of all patients with prevalent lung cancer, 36.0% resided in isolated or small rural areas, and 41.0% resided in the highest two quintiles for ADI. There were 4616 lung cancer deaths, and 26.5% occurred in patients aged 80 years or older. Prevalence of ever-smoking increased across categories of rurality from 49.2% (metropolitan) to 55.3% (small or isolated rural) (*P* < .001) and increased across ADI quintile from 46.9% (quintile 1) to 57.5% (quintile 5) (*P* < .001).

**Table 1.** Characteristics of the persons in the census, prevalent lung cancer cases, and lung cancer deaths in Maine

Characteristic	Person-years census data, No. (%)	Prevalent lung cancer cases, No. (%)	Lung cancer deaths, No. (%)
Age, y			
20-59	3 544 440 (70.4)	1051 (12.7)	747 (16.2)
60-69	767 170 (15.2)	2021 (24.4)	1262 (27.3)
70-79	428 870 (8.5)	2877 (34.7)	1385 (30.0)
≥80	295 400 (5.9)	2348 (28.3)	1222 (26.5)
Sex			
Female	2 603 315 (51.7)	4219 (50.8)	2080 (45.1)
Male	2 432 565 (48.3)	4078 (49.2)	2536 (54.9)
Rurality			
Metro	1 659 150 (32.9)	2495 (30.1)	1347 (29.2)
Large rural	1 802 410 (35.8)	2815 (33.9)	1604 (34.7)
Small/Isolated rural	1 574 320 (31.3)	2987 (36.0)	1665 (36.1)
Area deprivation index quintile			
1	823 355 (16.3)	1152 (13.9)	603 (13.1)
2	1 325 455 (26.3)	1980 (23.9)	1083 (23.5)
3	1 094 965 (21.7)	1766 (21.3)	960 (20.8)
4	1 100 380 (21.9)	1973 (23.8)	1142 (24.7)
5	691 725 (13.7)	1426 (17.2)	828 (17.9)

Lung cancer prevalence and mortality were positively associated with increasing rurality in crude analyses (Table 2). However, these associations did not persist in multivariable analyses adjusting for age, sex, and smoking.

**Table 2.** Rate ratios for lung cancer prevalence and mortality according to rurality

Rurality category	Crude (unadjusted) RR (95% CI)	Age and sex adjusted RR (95% CI)	Age, sex, and smoking adjusted* RR (95% CI)
Prevalence			
Urban	1.00 (Referent)	1.00 (Referent)	1.00 (Referent)
Large rural	1.04 (0.98 to 1.10)	1.05 (1.00 to 1.11)	1.01 (0.95 to 1.06)
Small/Isolated rural	1.26 (1.20 to 1.33)	1.04 (0.99 to 1.10)	0.98 (0.93 to 1.04)
Mortality			
Urban	1.00 (Referent)	1.00 (Referent)	1.00 (Referent)
Large rural	1.10 (1.02 to 1.18)	1.10 (1.02 to 1.18)	1.04 (0.97 to 1.13)
Small/Isolated rural	1.30 (1.21 to 1.40)	1.08 (1.01 to 1.16)	1.01 (0.94 to 1.09)

* *P*_trend_ for fully adjusted prevalence model = 0.54; *P*_trend_ for fully adjusted mortality model = 0.80. CI = confidence interval; RR = rate ratio.

Associations between lung cancer prevalence, mortality, and ADI are shown in Table 3. Lung cancer prevalence and mortality were positively associated with increasing ADI in models adjusted for age, sex, and smoking rates (prevalence rate ratio for ADI quintile 5 compared with quintile 1 = 1.41, 95% CI = 1.30 to 1.54, and mortality rate ratio = 1.59, 95% CI = 1.41 to 1.79). Multivariable analyses adjusting further for rurality did not change these associations (data not shown).

**Table 3.** Rate ratios for lung cancer prevalence and mortality according to area deprivation index

Area deprivation index quintile	Crude (unadjusted) RR (95% CI)	Age and sex adjusted RR (95% CI)	Age, sex, and smoking adjusted* RR (95% CI)
Prevalence			
1	1.00 (Referent)	1.00 (Referent)	1.00 (Referent)
2	1.07 (0.99 to 1.15)	1.25 (1.17 to 1.35)	1.25 (1.16 to 1.34)
3	1.15 (1.07 to 1.24)	1.34 (1.25 to 1.45)	1.32 (1.22 to 1.43)
4	1.28 (1.19 to 1.38)	1.38 (1.28 to 1.48)	1.35 (1.25 to 1.47)
5	1.47 (1.36 to 1.59)	1.44 (1.34 to 1.56)	1.41 (1.30 to 1.54)
Mortality			
1	1.00 (Referent)	1.00 (Referent)	1.00 (Referent)
2	1.12 (1.01 to 1.23)	1.30 (1.17 to 1.43)	1.29 (1.17 to 1.43)
3	1.20 (1.08 to 1.33)	1.37 (1.24 to 1.52)	1.37 (1.23 to 1.52)
4	1.42 (1.28 to 1.56)	1.52 (1.38 to 1.68)	1.51 (1.35 to 1.69)
5	1.63 (1.47 to 1.82)	1.60 (1.44 to 1.78)	1.59 (1.41 to 1.79)

* *P*_trend_ for fully adjusted prevalence model < .001; *P*_trend_ for fully adjusted mortality model < .001. CI = confidence interval; RR = rate ratio.

To explore how smoking might influence the observed associations, we examined the associations between smoking rates and both rurality and ADI. The proportion of people who were ever-smokers increased according to rurality from 49.2 (metropolitan) to 55.3 (small and isolated rural) (*P* < .001) and also increased according to ADI quintile from 46.9 (quintile 1) to 57.5 (quintile 5) (*P* < .001).

A majority of person-time for people in small and isolated rural areas were distributed in quintile 5 of ADI, indicating that a majority of the population in those areas experiences the highest degree of deprivation. Conversely, a majority of metropolitan dwellers person-time were distributed in ADI quintiles 1 to 4.

## Discussion

This study assessed the relationship between lung cancer prevalence and mortality and both rurality and socioeconomic deprivation in a rural state. Both lung cancer prevalence and mortality were strongly and positively associated with increasing ADI, a composite indicator of socioeconomic deprivation, and the association persisted after adjustment for age, sex, and area smoking rates. In contrast, lung cancer prevalence and mortality were not associated with increasing rurality after adjustment for age, sex, and smoking.

These findings update and extend those of Singh et al. (16), who reported that lung cancer mortality was associated with both socioeconomic deprivation and rurality, although the association with deprivation was stronger. Notably, the analyses of Singh et al. adjusted for age but not sex or smoking. Our study showed that adjusting for sex and smoking makes the association between lung cancer prevalence and mortality and rurality nonstatistically significant. This finding suggests that smoking may drive some of the previously reported rural disparities in cancer prevalence and mortality or may be an important confounding variable that has not been fully accounted for in prior studies.

Overall, our study suggests that socioeconomic deprivation, and not rurality, has an independent effect on lung cancer prevalence and mortality. Although the cross-sectional nature of our study limits strong inferences about causality, our data suggest the need for further research to elucidate how socioeconomic deprivation influences lung cancer outcomes. More research is also needed to confirm our findings and to elucidate how area deprivation relates to rurality and how these and other factors might interact.

Our study had several limitations. It focused on a single state with a large rural population, and its findings need to be replicated in other areas and populations. However, the socioeconomic diversity of rural communities in Maine may have enabled us to disentangle the independent effects of socioeconomic deprivation and rurality. The overall design of our study was cross-sectional and ecological. This limits our ability to make inferences about causation. However, studies of area-level health behaviors and disease are relevant for local population health interventions such as community-directed smoking prevention and cessation efforts. Claims analyses are limited to examining care that was received and are susceptible to ascertainment bias when care access is limited (eg, in rural or high-deprivation areas); people who did not seek care or were uninsured may have thus been underrepresented in our data. We were only able to examine prevalent (not incident) lung cancer cases in this analysis of claims, and we could not determine which cases were new, ongoing management of cases that may have been diagnosed before 2012 or recurrent diagnoses. Additionally, smoking prevalence and deprivation measures were estimated at the area level rather than at the individual level; thus, some individuals may have been higher income in low resource areas or nonsmokers in high smoking areas. Also, we used recent smoking prevalence data for this analysis, when the exposure period putting people at risk of lung cancer would have been decades prior in most cases, and the area smoking patterns may have changed over time. Our use of BRFSS data made it necessary to assign ADI to five-digit zip codes rather than the census-block level, which has been shown to be more robust (20). Finally, we conducted our analysis data during a time period when lung cancer screening with low-dose computed tomography was only beginning to be recommended and implemented (24). Replicating this study after more widespread use of low-dose computed tomography will be informative.

Our work has clinical and public health implications. Rural areas bear a disproportionate and increasing share of the overall burden of lung cancer in the United States (2), and there is growing interest in understanding and addressing these disparities. Our findings suggest that rurality itself, as a geographic phenomenon, may not be the critical factor or source of the disparity. Instead, the fact that rural communities, on average, experience greater economic deprivation may account for much of the rural-urban difference observed nationally and within states. If these findings are confirmed, the most strategic approach to reducing the burden of lung cancer and other malignancies may be to target prevention, screening, and treatment efforts toward communities that are socioeconomically deprived. Our findings would also suggest the need for more fundamental, systemic interventions to address this deprivation. Specifically, this could mean working with local communities to understand their health needs assessment reports and the opportunities they see to reach vulnerable populations. Additionally, developing multisector partnerships to effectively address social determinants of health such as joblessness, lack of reliable transportation, food insecurity, and poor housing could help support overall population health improvement.

Because smoking is the strongest risk factor for lung cancer and a number of other diseases, efforts to prevent people from starting to smoke, and tobacco treatment for current smokers, remain critical (25). Rural communities are experiencing growing disparities in smoking rates among adults (5) and youth (26) that have been driven by more rapid declines in smoking among residents of urban places. Programs to control tobacco that are already implemented are projected to reduce lung cancer rates going forward (27). However, a detailed review of tobacco prevention and control in the United States suggests that rural residents are more likely to live in states with fewer prevention policies and in communities without robust local tobacco-control provisions (28). Also, provider-based tobacco control and lung cancer screening programs will need to ensure active outreach to the more socioeconomically deprived areas to reach people who are less likely to access primary care and cancer (5) screening in general. This may be more challenging in rural states because of geographic isolation and shortages in primary care.

In conclusion, our study found that socioeconomic deprivation is associated with lung cancer prevalence and mortality, independent of smoking, whereas rurality is not. At the same time, both rural communities and socioeconomically disadvantaged communities have higher rates of smoking, although the interrelatedness of rurality, low SES, and smoking warrants further investigation. Our findings suggest that lung cancer prevention, screening, and treatment efforts should target populations with socioeconomic deprivation, rather than rurality per se. Additionally, efforts to improve access to treatment for those who are diagnosed with lung cancer are important to reduce morbidity and mortality. Future research should seek to better understand whether elevated rural smoking rates are a function of lower SES and should examine patient-level risk factors such as smoking history and access to medical care, including lung cancer screening.

## Supplementary Material

pkaa011_Supplementary_Data

## Notes

The study sponsors were not involved in any aspect of this research and did not participate in the decision to submit the manuscript for publication.

The authors thank the Maine Lung Cancer Coalition. We also thank the Maine Health Data Organization for its work aggregating the claims data.

Disclaimer: None of the authors have conflicts of interest or financial disclosures.
