# Supplementary material for: Area Deprivation Index and Rurality in Relation to Lung Cancer Prevalence and Mortality in a Rural State
Source: JNCI Cancer Spectr. 2020 Mar 7;4(4):pkaa011. doi: 10.1093/jncics/pkaa011 (PMC7353952; doi:10.1093/jncics/pkaa011)
Supplement: pkaa011_Supplementary_Data [file pkaa011_supplementary_data.pdf]

Supplementary Table 1

ICD10 Diagnosis Codes and Description			
C34.00	-	C34.02	Malignant neoplasm of main bronchus
C34.10	-	C34.12	Malignant neoplasm of upper lobe, bronchus or lung
C34.2			Malignant neoplasm of middle lobe, bronchus or lung
C34.30	-	C34.32	Malignant neoplasm of lower lobe, bronchus or lung
C34.80	-	C34.82	Malignant neoplasm of overlapping sites of bronchus and lung
C34.90	-	C34.92	Malignant neoplasm of unspecified part of bronchus or lung

ICD9 Diagnosis Codes and Description	
162.0	Malignant neoplasm trachea
162.2	Malignant neoplasm main bronchus
162.3	Malignant neoplasm upper lobe lung
162.4	Malignant neoplasm middle lobe lung
162.5	Malignant neoplasm lower lobe lung
162.8	Malignant neoplasm bronchus/lung NEC
162.9	Malignant neoplasm bronchus/lung NOS
